# Physical activity and risk of gallstone disease: A Mendelian randomization study

**DOI:** 10.3389/fgene.2022.943353

**Published:** 2022-12-06

**Authors:** Qilin Qian, Han Jiang, Bingyue Cai, Dingwan Chen, Minmin Jiang

**Affiliations:** ^1^ School of Public Health, Hangzhou Medical College, Hangzhou, China; ^2^ Key Laboratory of Pollution Exposure and Health Intervention of Zhejiang Province, Shulan International Medical College, Zhejiang Shuren University, Hangzhou, China

**Keywords:** physical activity, gallstone disease, Mendelian randomization analysis, casual association, genetic epidemiology

## Abstract

**Objective:** Given the association between physical activity and the reduced risk of gallstone disease as suggested in observational studies, a Mendelian randomization study was conducted to evaluate the causal nature of this association in genetic epidemiology.

**Study:** Including self-reported and accelerometer-based physical activity traits, the independent genetic variants associated with physical activity were selected from the corresponding genome-wide association studies as instrumental variables. The summary-level data for gallstone disease were sourced from the UK Biobank (7,682 cases and 455,251 non-cases) and FinnGen consortium (23,089 cases and 231,644 non-cases). Then, two-sample Mendelian randomization analysis was conducted. Inverse-variance weight (IVW), weighted median, and Mendelian randomization–Egger regression were determined through Mendelian randomization analyses. To ensure the robustness of the results, sensitivity analyses were also carried out in the study.

**Results:** The negative causality between the genetically predicted accelerometer-based “average acceleration” physical activity and the risk of gallstone disease was suggested in the UK Biobank study (*p* = 0.023, OR = 0.93, 95% CI: 0.87–0.99), and accelerometer-based “overall activity” physical activity and the risk of gallstone disease in the UK Biobank study (*p* = 0.017, OR = 0.38, 95% CI: 0.17–0.84). With accelerometer-based “average acceleration” physical activity negatively correlated with gallstone disease in the FinnGen consortium data (*p* = 0.001, OR = 0.94, 95% CI: 0.90–0.97). As for self-reported moderate-to-vigorous physical activity, however, there was no causality observed in both pieces of data.

**Conclusion:** Our studies provide the evidence suggesting a casual association between physical activities and gallstone disease through analysis of genetic data. As indicated by the research results, there is a possibility that a higher level of physical activities could mitigate the risk of gallstone disease.

## Introduction

As one of the most common diseases worldwide, gallstone disease (GSD) affects approximately 10%–20% adult population globally and the proportion of patients developing symptoms or complications surpasses 20% (Portincasa et al., 2006; Lammert et al., 2016). Generally, cholelithiasis can be classified into two types, including cholesterol and pigment types, and the most frequent form of GSD is cholesterol GSD. The recurrence of GSD and severe complications (e.g., cholecystitis, cholangitis, and pancreatitis) would have an impact on the health status of patients by causing gastrointestinal problems or chronic diseases (e.g., diabetes, cardiovascular diseases, and cancer) even after cholecystectomy (Shaheen et al., 2006; Wirth et al., 2020). Due to various factors such as overly nutritious diets, sedentary lifestyle, and aging, the incidence of GSD has shown an upward trend consistently in recent years (Lammert et al., 2016; Murphy et al., 2020; Wirth et al., 2020). As reported, GSD incurs high costs and increases the burden placed on the whole society. According to the relevant statistics, more than 65 billion is spent annually, given over 600,000 hospitalizations and approximately 1.8 million ambulatory care visits in the US (Shaffer, 2006). Previously, it was demonstrated in some studies that the formation of GSD is attributable to various factors, including a complex interrelationship between various genetic, environmental, and lifestyle factors (Stokes et al., 2011; Shabanzadeh, 2018). In spite of this, the root cause of GSD remains incompletely understood. Therefore, it is necessary to identify the risk factors for GSD formation and unravel its underlying mechanisms, which is essential for reducing the risk of GSD and lowering the financial burden placed on public health systems.

Over the past few decades, it has been suggested that a higher level of physical activity (PA) could be effective in preventing a variety of different chronic diseases (e.g., diabetes, stroke, cardiovascular diseases, and cancers) and in improving both physical and mental functions (Sattelmair et al., 2011; Mok et al., 2019). Currently, there are many observational studies indicating that PA is critical to the prevention of GSD (Chen et al., 2014; Zhang et al., 2017; Ryu et al., 2018; Kwon et al., 2020). Nevertheless, the causal association was not determined in these observational studies due to potential biases, such as causal conclusion confounding, reverse causation, and measurement error. For example, dietary habits are a potential confounder because it makes an impact on the risk of GSD (Davidovic et al., 2011). For reverse causation, low physical activity levels may be the consequence, rather than the cause of GSD, making it difficult to determine a causal association between the two. In addition, much of traditional observational studies come from self-reports; are susceptible to recall bias and social expectations bias; and subject to measurement error, for instance, people may report higher levels of PA in order to show they are healthy. Considering the weaknesses of regular observational studies, whether the observed association between PA and GSD reflects true causation or is confounded remains unclear.

In respect of the research on causality of risk factors and diseases, Mendelian randomization (MR) has received increasing popularity as a novel solution (Wang et al., 2019). Following the law of independent assortment, genetic variants are randomly allocated at the time of conception (Davey Smith and Hemani, 2014; Yarmolinsky et al., 2018). Therefore, they can be used to indicate the effect of the exposure that cannot be modified by the outcome (Burgess et al., 2013). With genetic variants as instrumental variables (IVs) for exposure, MR can prevent reverse causation and most residual confounding, thus strengthening the causal inference (Smith and Ebrahim, 2003). Recently, MR analysis has been increasingly relied on to explore the causal relationship between PA and diverse cancers (Papadimitriou et al., 2020), psychiatric disorders (Papiol et al., 2021), and cardiovascular diseases (Zhuang et al., 2020). Up to now, however, there is still no MR analysis conducted on the correlation between PA and GSD. Therefore, this paper aimed to reveal the potential causal association between the level of PA and the risk of cholesterol GSD through a MR study.

## Materials and methods

### Study design

**FIGURE 1:**
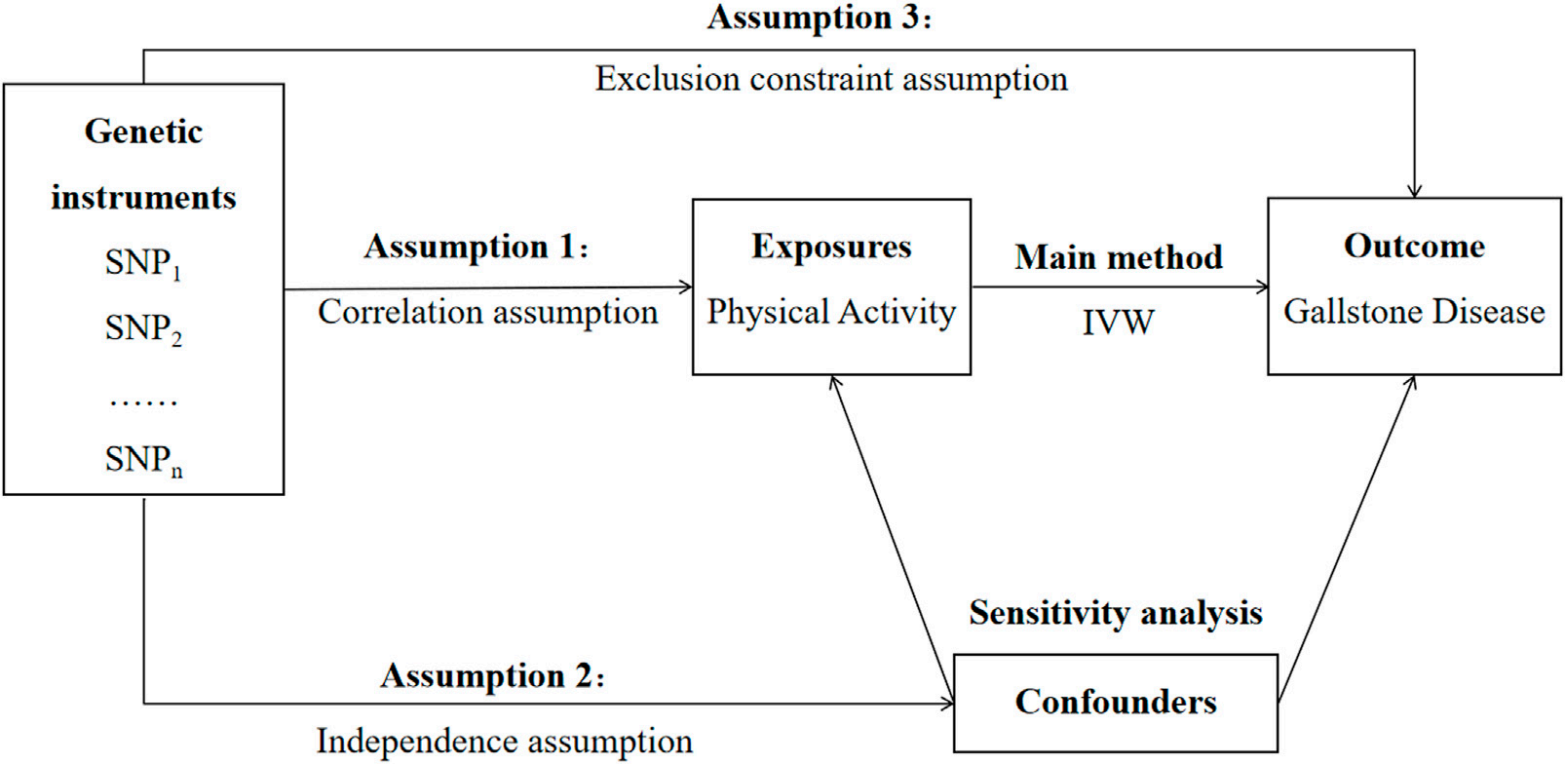
Study design overview. Abbreviation: SNP, single-nucleotide polymorphism; IVW, inverse-variance weighted.

There are three core assumptions that should be satisfied for a genetic variant to be a valid IV in MR analysis (Figure 1). First, it is a necessity for the selected genetic variant to be closely correlated with the exposure under study. Second, this genetic variant is supposed not to have any association with any confounding factors. Third, not only does this genetic variant have no common cause to share with the outcome, it also affects the outcomes through no other ways than the exposure.

### Data source for physical activities

The summary-level genetic data for PA were sourced from two published genome-wide association studies (GWAS) conducted in the UK Biobank, involving 91,105 participants of one GWAS and 377,234 cases of the other (Doherty et al., 2018; Klimentidis et al., 2018). As a population-based prospective cohort study, the UK Biobank study was conducted after the recruitment of over a half-million participants who were 40–69 years old residing in the UK (Fry et al., 2017). As for the data of the PA phenotypes selected in this study, they included self-reported moderate-to-vigorous PA, accelerometer-measured “average acceleration” PA, and accelerometer-measured “overall activity” PA. Self-reported moderate-to-vigorous PA was acquired through the International Physical Activity Short-Form Questionnaire. It was calculated by multiplying the total number of minutes of moderate and vigorous physical activity on a weekly basis by eight, corresponding to the metabolic equivalents (Guo et al., 2019). Two measures, overall acceleration average and average acceleration, were obtained from 1,000 participants wearing wrist-worn accelerometers among the populations under study (Guo et al., 2019). The data less than 72 h or not completed every hour in a 24-h cycle and other outliers were excluded. The accelerometer-measured “overall activity” levels were treated as average vector magnitude for each 30-s epoch (Doherty et al., 2017).

### Genetic instrument selection

Initially, the PLINK clumping method (*r* ^2^ = 0.001 and clumping distance = 10,000 kb) was applied to select a total of 19 single-nucleotide polymorphisms (SNPs) associated with self-reported moderate-to-vigorous PA and eight SNPs associated with accelerometer-measured “average acceleration” PA at the genome-wide significance level (*p* < 5 × 10^–8^) from the GWAS(24). Then, five SNPs associated with accelerometer-measured “overall activity” PA were also selected from the GWAS (Doherty et al., 2018). Linkage disequilibrium (LD) among SNPs for each risk factor was calculated based on a 1,000-genome LD reference panel (European population). If the summary statistics for specifically PA-related SNPs were unavailable in the outcome GWAS, SNPs in high linkage disequilibrium (*r* ^2^ > 0.80) would be adopted as proxy instruments. As IVs can only affect outcome through the exposure based on the primary hypothesis of MR analysis, we used the PhenoScanner website to test the pleiotropic effects of the selected IVs. It was found that none of the SNPs for self-reported moderate-to-vigorous PA, accelerometer-measured “average acceleration” PA, and accelerometer-measured “overall activity” PA showed association with potential confounders or gallstones (Supplementary Table S1). After evaluation was conducted on the potential pleiotropic effects for SNPs, 19, eight, and five SNPs were treated as IVs, respectively, for self-reported moderate-to-vigorous PA, accelerometer-measured “average acceleration” PA, and accelerometer-measured “overall activity” PA, respectively.

### Data source for gallstone disease

The cases with gallstone disease were defined in accordance with International Classification of Diseases, 10th Revision (ICD-10), and code K80. Cholesterol gallstone disease was caused by the disturbance of biliary cholesterol homeostasis. Two cohorts, the UK Biobank study and the FinnGen consortium data, were applied as the data source for cholesterol GSD, so as to explore the correlation between PA-associated SNPs and cholesterol GSD. Sourced from the UK Biobank cohort for use in this study, the data involved 462,933 participants (7,682 cases and 455,251 controls) of European ancestry. In addition, the R6 release of the FinnGen consortium dataset was used in this paper, involving 254,733 participants (23,089 cases and 231,644 controls). Those individuals with ambiguous gender, high genotype missingness (>5%), excess heterozygosity (±4SDs), and non-Finnish ancestry were also excluded from the FinnGen consortium data.

### Statistical analysis

Herein, inverse-variance weighted (IVW) method was used as the primary method of MR analysis because it allowed each SNP to have different mean effects for obtaining the MR estimates as to the causal effect of exposure on the relevant results based on the two-sample GWAS summary data (Hartwig et al., 2016). Odds ratios (ORs) and the corresponding confidence intervals (CIs) of GSD were scaled to a 1-SD increase in self-reported moderate-to-vigorous PA (MET-minutes/week), a 1-SD increase of accelerometer-measured “average acceleration” PA, and a 1-SD increase of accelerometer-measured “overall activity” PA in the UK Biobank study.

Notably, it is possible for the introduction of multi-validity instrumental variables to result in biased results. To further assess the research results for their robustness, a series of sensitivity analyses were conducted, including the weighted median (WM) method (Bowden et al., 2016), MR–Egger regression (Bowden et al., 2015), MR pleiotropy residual sum and outlier (MR-PRESSO) (Verbanck et al., 2018), and robust adjusted profile score (RAPS) (Zhao et al., 2018). Even though when up to 50% of the genetic variation comes from invalid IV, consistent estimates can still be made by the WM model (Bowden et al., 2016). MR–Egger regression analysis was carried out to assess and correct the horizontal pleiotropic effects, where the *p*-value for the intercept >0.05 indicates no pleiotropy (Bowden et al., 2015). The MR-PRESSO approach can be adopted to detect outliers and make estimates for outliers in the context of IVW linear regression. In addition, Cochran’s Q test was performed to assess the statistical heterogeneity among SNPs for IVW estimates. Furthermore, we eliminated each single SNP at a time to detect whether the risk estimates of PA on GSD has basically remained consistent, which was referred to as leave-one-out analysis. It was conducted to evaluate the impact of individual SNPs on the summary estimates, so as to detect the presence of pleiotropy. For part of the exposure and outcome data, both of which were sourced from the UK Biobank study, there was a possibility of large sample overlap between them. For this reason, the F statistic was calculated to measure the strength of instrument in the study (Burgess et al., 2011). RAPS was used to give a more robust inference for MR analysis results, which was robust to both systematic and idiosyncratic pleiotropy (Wu et al., 2020).

These statistical models were operated using the TwoSampleMR (version 0.5.2) (Hemani et al., 2018) and MR-PRESSO (version 1.0) (Verbanck et al., 2018) packages in R software (version 3.6.3), with all estimates two-sided at a significance level of ≤0.05.

## Results

Supplementary Table S2 presents a summary of three PA phenotypes and characteristics of the SNPs treated as instrumental variables. The F statistics for all PA genetic instruments were >10, which indicates no weak instrument bias. Supplementary Table S3 shows the association of genome-wide significant SNPs for self-reported moderate-to-vigorous physical activity with gallstones. Supplementary Table S4 shows the association of genome-wide significant SNPs for accelerometer-measured “average acceleration” physical activity with gallstones. Supplementary Table S5 shows the association of genome-wide significant SNPs for accelerometer-measured “overall activity” physical activity with gallstones.

**FIGURE 2:**
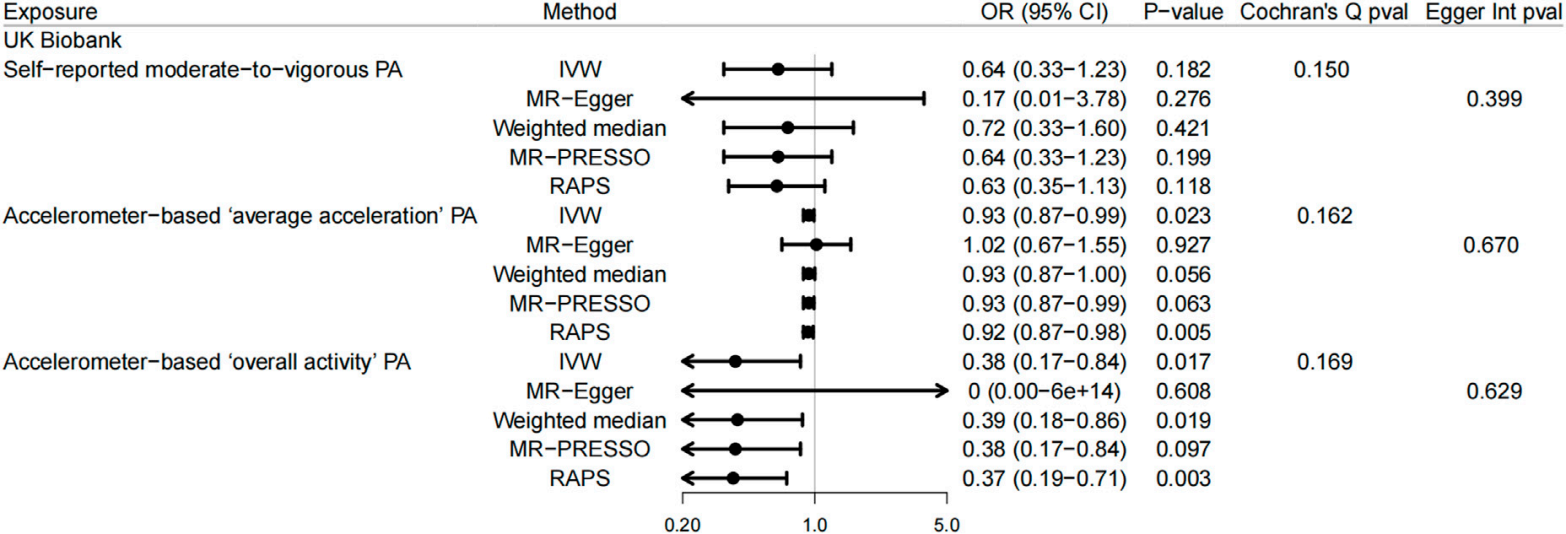
Mendelian randomization estimates from each method of assessing the causal effects of physical activity on gallstone disease in the UK Biobank study. Abbreviation: UKB, UK Biobank; PA, physical activity; IVW, inverse-variance weighted; WM, weighted median; MR-PRESSO, MR pleiotropy residual sum and outlier; RAPS, robust adjusted profile score; OR, odds ratio; CI, confidence interval.

**FIGURE 3:**
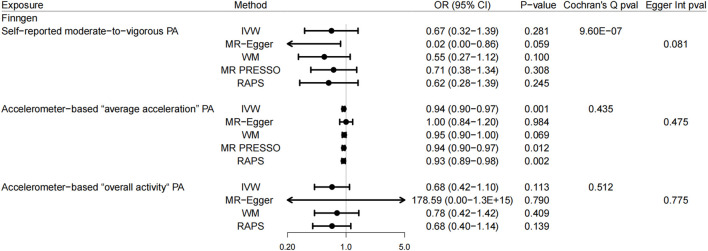
Mendelian randomization estimates from each method of assessing the causal effects of physical activity on gallstones disease in the FinnGen consortium data. Abbreviation: PA, physical activity; IVW, inverse-variance weighted; WM, weighted median; MR-PRESSO, MR pleiotropy residual sum and outlier; RAPS, robust adjusted profile score; OR, odds ratio; CI, confidence interval.

In the UK Biobank study, there was a correlation suggested between genetically predicted accelerometer-based “average acceleration” PA and the risk of GSD (*p* = 0.023, OR = 0.93, 95% CI: 0.87–0.99, using IVW) (Figure 2). A similar result was found here in the FinnGen consortium data for accelerometer-based “average acceleration” PA (*p* = 0.001, OR = 0.94, 95% CI: 0.90–0.97, using IVW) (Figure 3). While accelerometer-based “overall activity” PA was associated only with GSD in the UK Biobank data (*p* = 0.017, OR = 0.38, 95% CI: 0.17–0.84, using IVW) (Figure 2) rather than in the FinnGen consortium data. With regard to the correlation between self-reported moderate-to-vigorous PA and GSD, there was no convincing evidence found to suggest any causality, whether in the UK Biobank study or the FinnGen consortium data. These findings for self-reported moderate-to-vigorous PA, accelerometer-based “average acceleration” PA, and accelerometer-based “overall activity” PA in relation to GSD were basically coherent in sensitivity analyses. Despite the detected evidence of mild heterogeneity in Cochran’s Q test in FinnGen consortium data, it was still revealed by leave-one-out analysis (Supplementary Figure S1) that the results were not substantially affected by a specific SNP.

## Discussion

To the best of our knowledge, this study is the first analysis, with a large sample size of participants involved in the MR analyses, to explore whether there is any link between PA and the risk of GSD. According to the data shown in the results, PA is suspected to have an inverse correlation with the risk of GSD, which is largely consistent with the findings of most previous observational studies.

It has now been suggested in plenty of observational studies that there was an inverse association between PA and GSD. As supported by a cross-sectional case-control study of Chinese women, GSD risk was positively associated with PA (Hou et al., 2009). In addition, the multivariate analysis conducted in an observational study with 8,908 participants demonstrated that the absence of PA was one of the independent influencing factors for GSD among males (Kwon et al., 2020). As revealed by Storti et al. in some cohort studies, PA could play a role in preventing GSD for post-menopausal women (Storti et al., 2005). While Leitzmann et al. discovered a similar consequence among 45,813 men (Leitzmann et al., 1998). In another population-based prospective cohort study involving 147,237 participants, it was found out that the incidence of GSD was lower among the health-enhancing physically active group, with both increased participation in physical activity and reduced sitting time, suspected to be crucial for mitigating the risk of GSD separately (Ryu et al., 2018). Including six case-control studies and 13 cohort studies, a systematic review and meta-analysis suggested a 15% reduction in the risk of GSD among the most physically active individuals compared to the least active individuals, regardless of their gender (Zhang et al., 2017). However, there is another study which was inconclusive about the association between PA and GSD as Kono et al. (1995) failed to observe any correlation between PA and gallstones in the analysis conducted among 2,228 men aged 49–55 years receiving a retirement health examination at three hospitals of the Japan Self-Defense Forces between 1991 and 1992 (40). These contradictory results were possibly attributable to the small sample size, the limitation of specific population, or the lack of accuracy in the methods used to evaluate PA [(Chen et al., 2014)].

There are various potential mechanisms that can be used to illustrate the inverse association between PA and GSD. As gallstone development is suspected to be closely linked to biliary cholesterol supersaturation (Carey and Lamont, 1992), physical activities could contribute to changing the cholesterol uptake from circulation, cholesterol biosynthesis in the liver, or the catabolism level of cholesterol to bile acids, thus having a significant impact on GSD (2). In addition, PA can possibly exhibit a protective effect by enhancing gallbladder motility, reducing biliary stasis (Wilund et al., 2008), and influencing many pancreatic and gastrointestinal hormones (Sullivan et al., 1984), thus playing a protective role in GSD. From a different perspective, PA can also exhibit a prokinetic effect on the gut, which makes a difference to intestinal transit (Marcus and Heaton, 1986). Slow colonic transit can be one of the risk factors for gallstones. PA could reduce the level of deoxycholic acid and cholesterol saturation in bile by accelerating colonic transit (Marcus and Heaton, 1986) to enable the normal physiological functioning of the gallbladder. Furthermore, it has been demonstrated in some studies that those who tend to do more physical activities may be higher in the level of high-density lipoprotein cholesterol but lower in the level of triglyceride in serum (Seals et al., 1984; Baker et al., 1986). High-density lipoprotein cholesterol is inversely correlated with bile lithogenicity (Thornton et al., 1981). Triglycerides may secrete mucin hypersecretion and facilitate the formation of biliary cholesterol lithogenesis through the stimulation of gallbladder mucous membrane cells (Mingrone et al., 1988). Additionally, it has been suggested that impaired glucose tolerance and upper insulin levels can be the risk factors for gallstones. PA is effective in improving glucose tolerance and reducing insulin levels through the enhancement of glucose utilization (Rosenthal et al., 1983; Scragg et al., 1984). Insulin plays a vital role in activating the low-density lipoprotein receptors in hepatic (Chait et al., 1979) and 3-hydroxy-3-methylglutaryl coenzyme A reductase (Nepokroeff et al., 1974) and in suppressing 7a-hydroxylase activity (Subbiah and Yunker, 1984), which enhances the absorption of cholesterol by the liver. In this sense, the development of GSD can be effectively prevented by the improvement of glucose tolerance and the decrease in insulin levels.

There were both advantages and limitations for this study. MR analyses strengthen the inference of a causal relationship between PA and GSD. With the randomly allocated alleles assigned to offspring and basically fixed at the time of conception, the MR results were made less susceptible to confounding and reverse causation measured with error when compared to conventional observational studies. In addition to the self-reported PA, an investigation was also conducted into objectively measured PA, which was considered more accurate and reliable than self-reported measurements. Meanwhile, various PA phenotypes (both binary and continuous variables) were also analyzed to assess the association between PA and the risk of GSD in a more thorough way. Moreover, two independent populations and sensitivity analyses were conducted to test the associations, which ensures more comprehensive and consistent findings. In addition, the population bias was avoided as the populations under study were all individuals of European ancestry.

However, there are several limitations worth noting. Suspected to be the main limitation in the study, horizontal pleiotropy can cause the outcome to be affected by genetic instruments through ways other than physical activities. Herein, no single SNP has any significant impact on the outcomes according to leave-one-out analysis, which is conducive to circumventing horizontal pleiotropy. Second, this research involves only European individuals, which means our consequences might not be suitable for extension to other populations. It is thus essential to verify the outcomes in other populations. Third, since the exposure and outcome data are sourced partially from the same GWAS, it might cause overfitting of the model and make the causal estimates lean toward observational associations in case of too many overlapping samples. However, the F statistic>10 reduces this likelihood to a significant extent. With a tiny fraction of the phenotypic variability explained by the genetic variants for accelerometer-assessed PA, a wide range of data sources are used in our study to address this problem. Finally, given the differences in allele frequencies between Finnish and other EU ethnic groups, this may induce some bias in the casual associations, and the summary-level data used in this study could not help conduct the stratified analysis by age and sex.

To sum up, this study provides the MR evidence suggesting a causal protective role of PA in GSD. It is demonstrated that the augmentation of physical activities may play a part in preventing GSD, which may provide the significant details about public health recommendations. Therefore, further research is required to unravel the underlying mechanisms between these two conditions, which contribute new strategies to disease prevention and health promotion.

## Data Availability

The original contributions presented in the study are included in the article/Supplementary Material; further inquiries can be directed to the corresponding author.
