# Supplementary material for: Physical activity and risk of gallstone disease: A Mendelian randomization study
Source: Front Genet. 2022 Dec 6;13:943353. doi: 10.3389/fgene.2022.943353 (PMC9763559; doi:10.3389/fgene.2022.943353)
Supplement: Supplementary file 1 [file Table1.DOCX]

Supplementary Table 1. Association (*P*<5x10^-8^) of the SNPs used as candidate genetic instruments for physical activity with confounders or gall stones

| SNP | CHR | Position (hg19/b37) | | Trait | Exclude from MR |
| --- | --- | --- | --- | --- | --- |
| **Self-reported moderate-to-vigorous PA** |  |  | |  |  |
| rs2942127 | 1 | 204420067 | | None | No |
| rs1974771 | 2 | 54278543 | | None | No |
| rs2114286 | 3 | 41194283 | | None | No |
| rs877483 | 3 | 53846741 | | None | No |
| rs2035562 | 3 | 85056521 | | None | No |
| rs1972763 | 4 | 159860563 | | None | No |
| rs77742115 | 5 | 18330424 | | None | No |
| rs1186721 | 7 | 34974602 | | None | No |
| rs7804463 | 7 | 133447651 | | None | No |
| rs1043595 | 7 | 128410012 | | None | No |
| rs7791992 | 7 | 50237784 | | None | No |
| rs921915 | 7 | 50228581 | | None | No |
| rs2988004 | 9 | 37044388 | | None | No |
| rs7326482 | 13 | 54037803 | | None | No |
| rs10145335 | 14 | 98547748 | | None | No |
| rs4886868 | 15 | 74353561 | | None | No |
| rs12912808 | 15 | 95292223 | | None | No |
| rs429358 | 19 | 45411941 | | None | No |
| rs1921981 | 21 | 42422547 | | None | No |
| **Accelerometer-based ‘average acceleration’ PA** |  |  | |  |  |
| rs34517439 | 1 | | 78450517 | None | No |
| rs6775319 | 3 | | 18758501 | None | No |
| rs9293503 | 5 | | 87948962 | None | No |
| rs12522261 | 5 | | 152054825 | None | No |
| rs11012732 | 10 | | 21830104 | None | No |
| rs148193266 | 11 | | 104528681 | None | No |
| rs56194509 | 17 | | 43844559 | None | No |
| rs59499656 | 18 | | 40768309 | None | No |
| **Accelerometer-based ‘overall activity’ PA** |  | |  |  |  |
| rs2696625 | 17 | | 44326864 | None | No |
| rs564819152 | 10 | | 21820650 | None | No |
| rs59499656 | 18 | | 40768309 | None | No |
| rs6775319 | 3 | | 18758501 | None | No |
| rs6895232 | 5 | | 152039421 | None | No |

Abbreviation: SNP, single-nucleotide polymorphism; CHR, chromosome.

Supplementary Table 2. Physical activity related SNPs from the GWAS used as genetic instruments in the MR analysis

| SNP | CHR | Position (hg19/b37) | EA | OA | EAF | BETA | SE | | *P*-value | R^2^ | F statistic |
| --- | --- | --- | --- | --- | --- | --- | --- | --- | --- | --- | --- |
| **Self-reported moderate-to-vigorous PA** |  |  |  |  |  |  |  | |  |  |  |
| rs2942127 | 1 | 204420067 | G | A | 0.82 | 0.016 | 0.003 | | 3.3e-08 | 0.00008 | 28.4 |
| rs1974771 | 2 | 54278543 | A | G | 0.10 | 0.021 | 0.004 | | 6.6e-09 | 0.00007 | 27.6 |
| rs2114286 | 3 | 41194283 | G | A | 0.53 | 0.012 | 0.002 | | 3.3e-08 | 0.00010 | 36.0 |
| rs877483 | 3 | 53846741 | T | C | 0.57 | 0.012 | 0.002 | | 4.0e-08 | 0.00010 | 36.0 |
| rs2035562 | 3 | 85056521 | G | A | 0.67 | 0.014 | 0.002 | | 3.9e-09 | 0.00013 | 49.0 |
| rs1972763 | 4 | 159860563 | C | T | 0.66 | 0.013 | 0.002 | | 3.3e-08 | 0.00011 | 42.2 |
| rs77742115 | 5 | 18330424 | C | T | 0.14 | 0.018 | 0.003 | | 9.6e-09 | 0.00010 | 36.0 |
| rs1186721 | 7 | 34974602 | A | G | 0.68 | 0.013 | 0.002 | | 4.4e-08 | 0.00011 | 42.2 |
| rs7804463 | 7 | 133447651 | T | C | 0.53 | 0.015 | 0.002 | | 1.2e-11 | 0.00015 | 56.2 |
| rs1043595 | 7 | 128410012 | G | A | 0.72 | 0.014 | 0.002 | | 4.3e-09 | 0.00013 | 49.0 |
| rs7791992 | 7 | 50237784 | C | A | 0.41 | 0.014 | 0.002 | | 5.7e-10 | 0.00013 | 49.0 |
| rs921915 | 7 | 50228581 | C | T | 0.59 | 0.014 | 0.002 | | 5.7e-10 | 0.00013 | 49.0 |
| rs2988004 | 9 | 37044388 | G | T | 0.44 | 0.013 | 0.002 | | 4.1e-09 | 0.00011 | 42.2 |
| rs7326482 | 13 | 54037803 | T | G | 0.62 | 0.013 | 0.002 | | 1.6e-08 | 0.00011 | 42.2 |
| rs10145335 | 14 | 98547748 | A | G | 0.25 | 0.014 | 0.003 | | 2.7e-08 | 0.00006 | 21.8 |
| rs4886868 | 15 | 74353561 | G | T | 0.59 | 0.012 | 0.002 | | 3.5e-08 | 0.00010 | 36.0 |
| rs12912808 | 15 | 95292223 | C | T | 0.15 | 0.018 | 0.003 | | 1.7e-08 | 0.00010 | 36.0 |
| rs429358 | 19 | 45411941 | C | T | 0.15 | 0.022 | 0.003 | | 6.1e-13 | 0.00014 | 53.8 |
| rs1921981 | 21 | 42422547 | G | A | 0.33 | 0.013 | 0.002 | | 3.8e-08 | 0.00011 | 42.2 |
| **Accelerometer-based ‘average acceleration’ PA** |  |  |  |  |  |  |  | |  |  |  |
| rs34517439 | 1 | 78450517 | C | A | 0.12 | 0.308 | | 0.056 | 4.4e-08 | 0.00033 | 30.2 |
| rs6775319 | 3 | 18758501 | A | T | 0.73 | 0.225 | | 0.041 | 3.5e-08 | 0.00033 | 30.1 |
| rs9293503 | 5 | 87948962 | T | C | 0.11 | 0.329 | | 0.059 | 2.1e-08 | 0.00034 | 31.1 |
| rs12522261 | 5 | 152054825 | G | A | 0.34 | 0.211 | | 0.038 | 3.9e-08 | 0.00034 | 30.8 |
| rs11012732 | 10 | 21830104 | A | G | 0.33 | 0.225 | | 0.039 | 5.4e-09 | 0.00037 | 33.3 |
| rs148193266 | 11 | 104528681 | C | A | 0.04 | 0.510 | | 0.092 | 3.1e-08 | 0.00034 | 30.7 |
| rs56194509 | 17 | 43844559 | G | T | 0.22 | 0.303 | | 0.044 | 5.0e-12 | 0.00052 | 47.4 |
| rs59499656 | 18 | 40768309 | T | A | 0.34 | 0.228 | | 0.038 | 2.4e-09 | 0.00040 | 36.0 |
| **Accelerometer-based ‘overall activity’ PA** |  |  |  |  |  |  | |  |  |  |  |
| rs2696625 | 17 | 44326864 | G | A | 0.77 | 0.037 | 0.005 | | 6.8e-14 | 0.00060 | 54.8 |
| rs564819152 | 10 | 21820650 | A | G | 0.68 | 0.028 | 0.005 | | 1.1e-08 | 0.00034 | 31.4 |
| rs59499656 | 18 | 40768309 | T | A | 0.66 | 0.028 | 0.005 | | 1.1e-08 | 0.00034 | 31.4 |
| rs6775319 | 3 | 18758501 | A | T | 0.27 | 0.027 | 0.005 | | 3.3e-08 | 0.00032 | 29.2 |
| rs6895232 | 5 | 152039421 | T | A | 0.66 | 0.027 | 0.005 | | 3.3e-08 | 0.00032 | 29.2 |

Abbreviation: SNP, single-nucleotide polymorphism; PA, physical activity; CHR, chromosome; EA, effect allele; OA, other allele; EAF, effect allele frequency; SE, standard error.

Supplementary Table 3. Association of genome-wide significant SNPs for self-reported moderate-to-vigorous physical activity with gall stones

| SNP | CHR | Position (hg19/b37) | EA | OA | BETA | SE | *P*-value |
| --- | --- | --- | --- | --- | --- | --- | --- |
| **UK Biobank** |  |  |  |  |  |  |  |
| rs877483 | 3 | 53846741 | C | T | 0.012 | 0.017 | 0.470 |
| rs7326482 | 13 | 54037803 | T | G | 0.006 | 0.017 | 0.720 |
| rs2854277 | 6 | 32628084 | C | T | 0.027 | 0.038 | 0.480 |
| rs7804463 | 7 | 133447651 | C | T | 0.022 | 0.016 | 0.170 |
| rs1186721 | 7 | 34974602 | A | G | 0.029 | 0.018 | 0.094 |
| rs2988004 | 9 | 37044388 | T | G | 0.004 | 0.016 | 0.810 |
| rs1921981 | 21 | 42422547 | G | A | 0.021 | 0.018 | 0.230 |
| rs429358 | 19 | 45411941 | T | C | 0.054 | 0.022 | 0.017 |
| rs921915 | 7 | 50228581 | C | T | 0.006 | 0.017 | 0.720 |
| rs2942127 | 1 | 204420067 | A | G | 0.053 | 0.021 | 0.013 |
| rs77742115 | 5 | 18330424 | T | C | 0.007 | 0.024 | 0.760 |
| rs2035562 | 3 | 85056521 | A | G | 0.018 | 0.017 | 0.310 |
| rs10145335 | 14 | 98547748 | A | G | 0.003 | 0.019 | 0.890 |
| rs4886868 | 15 | 74353561 | G | T | 0.011 | 0.017 | 0.520 |
| rs2114286 | 3 | 41194283 | A | G | 0.025 | 0.016 | 0.130 |
| rs1974771 | 2 | 54278543 | G | A | 0.006 | 0.027 | 0.830 |
| rs1043595 | 7 | 128410012 | A | G | 0.025 | 0.018 | 0.160 |
| rs1972763 | 4 | 159860563 | C | T | 0.018 | 0.017 | 0.290 |
| **Finngen** |  |  |  |  |  |  |  |
| rs10145335 | 14 | 98547748 | G | A | 0.0084 | 0.0178 | 0.638 |
| rs1043595 | 7 | 128410012 | G | A | 0.0075 | 0.0181 | 0.678 |
| rs1186721 | 7 | 34974602 | G | A | 0.0349 | 0.0165 | 0.034 |
| rs12912808 | 15 | 95292223 | C | T | 0.0315 | 0.0205 | 0.124 |
| rs1921981 | 21 | 42422547 | G | A | 0.0314 | 0.0165 | 0.058 |
| rs1972763 | 4 | 159860563 | C | T | 0.0201 | 0.0169 | 0.233 |
| rs1974771 | 2 | 54278543 | G | A | 0.0561 | 0.0208 | 0.007 |
| rs2035562 | 3 | 85056521 | A | G | 0.0116 | 0.0150 | 0.442 |
| rs2114286 | 3 | 41194283 | A | G | 0.0009 | 0.0151 | 0.953 |
| rs2942127 | 1 | 204420067 | G | A | 0.0337 | 0.0182 | 0.064 |
| rs2988004 | 9 | 37044388 | T | G | 0.0436 | 0.0153 | 0.004 |
| rs429358 | 19 | 45411941 | T | C | 0.0240 | 0.0196 | 0.221 |
| rs4886868 | 15 | 74353561 | T | G | 0.0109 | 0.0151 | 0.468 |
| rs7326482 | 13 | 54037803 | G | T | 0.0052 | 0.0154 | 0.737 |
| rs77742115 | 5 | 18330424 | T | C | 0.0100 | 0.0201 | 0.619 |
| rs7791992 | 7 | 50237784 | C | A | 0.0226 | 0.0150 | 0.133 |
| rs7804463 | 7 | 133447651 | T | C | 0.0151 | 0.0153 | 0.326 |
| rs877483 | 3 | 53846741 | T | C | 0.0003 | 0.0150 | 0.983 |

Abbreviation: SNP, single-nucleotide polymorphism; CHR, chromosome; EA, effect allele; OA, other allele; SE, standard error.

Supplementary Table 4. Association of genome-wide significant SNPs for accelerometer-measured ‘average acceleration’ physical activity with gall stones

| SNP | CHR | Position (hg19/b37) | EA | OA | BETA | SE | *P*-value |
| --- | --- | --- | --- | --- | --- | --- | --- |
| **UK Biobank** |  |  |  |  |  |  |  |
| rs59499656 | 18 | 40768309 | A | T | 0.021 | 0.017 | 0.230 |
| rs9293503 | 5 | 87948962 | T | C | 0.007 | 0.026 | 0.780 |
| rs6775319 | 3 | 18758501 | T | A | 0.043 | 0.018 | 0.018 |
| rs34517439 | 1 | 78450517 | A | C | 0.021 | 0.025 | 0.390 |
| rs12522261 | 5 | 152054825 | A | G | 0.000 | 0.011 | 0.990 |
| rs56194509 | 17 | 43844559 | T | G | 0.021 | 0.020 | 0.280 |
| rs11012732 | 10 | 21830104 | G | A | 0.049 | 0.017 | 0.004 |
| **Finngen** |  |  |  |  |  |  |  |
| rs11012732 | 10 | 21830104 | G | A | 0.0292 | 0.0119 | 0.014 |
| rs12522261 | 5 | 152054825 | A | G | 0.0048 | 0.0118 | 0.682 |
| rs148193266 | 11 | 104528681 | C | A | 0.0120 | 0.0312 | 0.700 |
| rs34517439 | 1 | 78450517 | A | C | 0.0428 | 0.0165 | 0.010 |
| rs56194509 | 17 | 43844559 | T | G | 0.0144 | 0.0200 | 0.472 |
| rs59499656 | 18 | 40768309 | A | T | 0.0073 | 0.0112 | 0.515 |
| rs6775319 | 3 | 18758501 | T | A | 0.0214 | 0.0117 | 0.067 |
| rs9293503 | 5 | 87948962 | C | T | 0.0186 | 0.0231 | 0.421 |

Abbreviation: SNP, single-nucleotide polymorphism; CHR, chromosome; EA, effect allele; OA, other allele; SE, standard error.

Supplementary Table 5. Association of genome-wide significant SNPs for accelerometer-measured ‘overall activity’ physical activity with gall stones

| SNP | CHR | | Position (hg19/b37) | | EA | OA | BETA | SE | | *P*-value | |
| --- | --- | --- | --- | --- | --- | --- | --- | --- | --- | --- | --- |
| **UK Biobank** | |  | |  |  |  |  | |  | |  |
| rs59499656 | | 18 | | 40768309 | T | A | -0.021 | | 0.017 | | 0.230 |
| rs6775319 | | 3 | | 18758501 | T | A | 0.043 | | 0.018 | | 0.018 |
| rs6895232 | | 5 | | 152047146 | A | T | 0.000 | | 0.016 | | 0.980 |
| rs564819152 | | 10 | | 21827796 | G | A | 0.047 | | 0.017 | | 0.006 |
| rs59499656 | | 18 | | 40768309 | T | A | -0.021 | | 0.017 | | 0.230 |
| **Finngen** | |  | |  |  |  |  | |  | |  |
| rs59499656 | | 18 | | 40768309 | A | T | 0.0217 | | 0.0152 | | 0.154 |
| rs6775319 | | 3 | | 18758501 | A | T | -0.0128 | | 0.0159 | | 0.421 |
| rs6895232 | | 5 | | 152039421 | T | A | -0.0208 | | 0.0161 | | 0.196 |

Abbreviation: SNP, single-nucleotide polymorphism; CHR, chromosome; EA, effect allele; OA, other allele; SE, standard error.


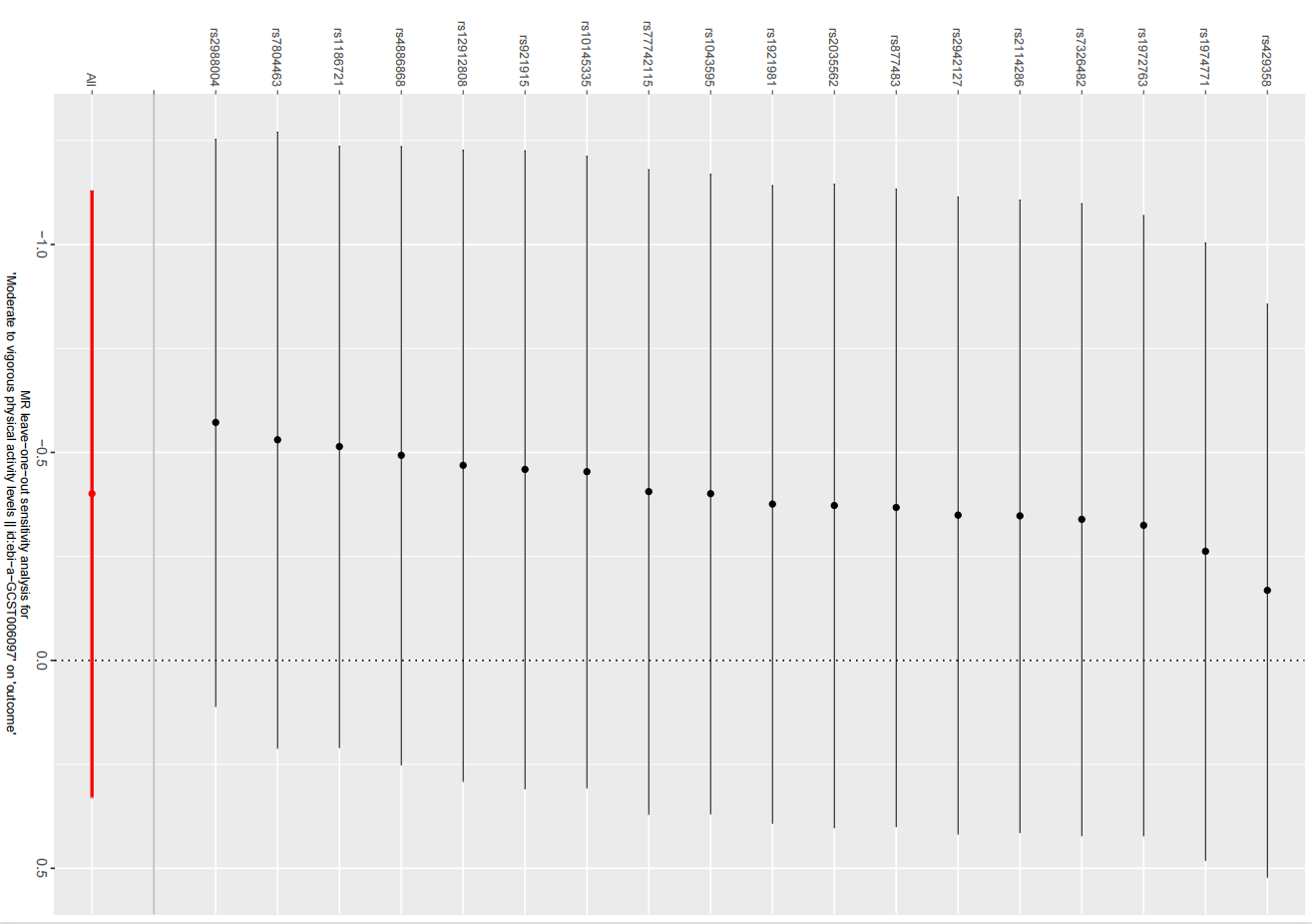


**Supplementary Figure 1.**
